# A Case of Brucellosis with Recurrent Attacks of Vasculitis

**DOI:** 10.1155/2016/5740589

**Published:** 2016-03-03

**Authors:** Pınar Korkmaz, Mehtap Kıdır, Nazlı Dizen Namdar, Ahmet Özmen, Cemile Uyar, Ayşe Nur Değer

**Affiliations:** ^1^Department of Infectious Diseases and Clinical Microbiology, Faculty of Medicine, Dumlupinar University, 43100 Kütahya, Turkey; ^2^Department of Dermatology, Faculty of Medicine, Dumlupinar University, 43100 Kütahya, Turkey; ^3^Department of Infectious Diseases and Clinical Microbiology, Evliya Celebi Training and Research Hospital, 43100 Kütahya, Turkey; ^4^Department of Pathology, Faculty of Medicine, Dumlupinar University, 43100 Kütahya, Turkey

## Abstract

Brucellosis is a zoonosis that affects several organs or systems. Skin involvement is nonspecific and it is reported to range between 0,4 and 17% of the patients with brucellosis. Here, we defined a 36-year-old female patient presented to our clinic with a clinical picture of recurrent attacks of vasculitis due to brucellosis for the first time. Skin involvement and vasculitic lesions as a finding of skin involvement are nonspecific in brucellosis. Therefore, in the regions like Turkey where brucellosis is endemic, brucellosis should be kept in mind necessarily in the differential diagnosis of vasculitis.

## 1. Introduction

Brucellosis is a zoonotic infectious disease caused by Gram-negative bacteria from the genus* Brucella* and almost all organs can be affected. Since brucellosis may mimic various multisystemic diseases and it may show a wide range of clinical polymorphism, this condition frequently causes misdiagnosis, delay in the treatment, and increase in complications due to the disease in brucellosis. The most commonly affected systems are locomotor, gastrointestinal, genitourinary, hematologic, cardiovascular, respiratory, and central nervous systems [1, 2]. Skin involvement was reported in 0,4 to 17% of the patients with brucellosis [3]. Here, we aimed to report the patient presented to our clinic with a clinical picture of recurrent attacks of vasculitis due to brucellosis.

## 2. Case Report

**Figure 1:**
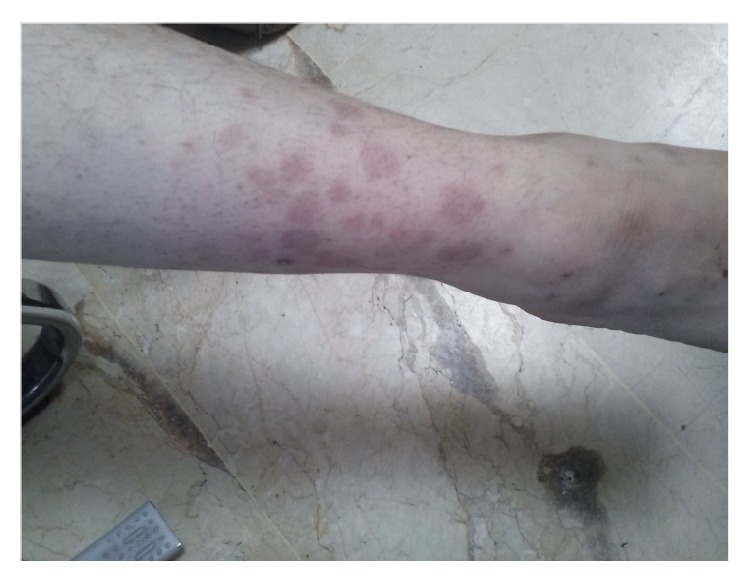
Nonblanchable maculopapular eruptions on the anterior parts of tibias.

**Figure 2:**
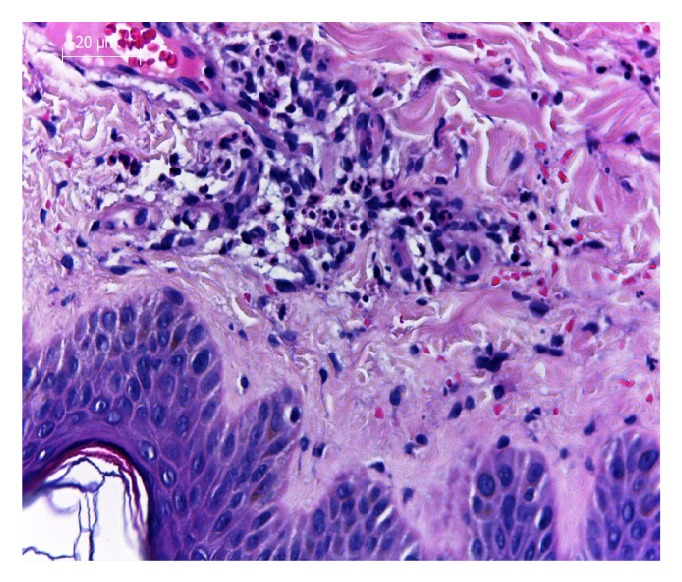
The walls of the small blood vessels are infiltrated by mixed inflammatory cells; skin biopsy showing leukocytoclastic vasculitis.

A 36-year-old female patient presented to dermatology clinic with a complaint of rash along the anterior parts of both lower legs for 2 weeks. An incisional skin biopsy was performed due to presence of widespread nonblanchable maculopapular eruptions on the anterior parts of tibias at the physical examination of the patient. Skin biopsy revealed leukocytoclastic vasculitis. The walls of the small blood vessels are infiltrated by mixed inflammatory cells (Figures 1 and 2). Treatment of vasculitis was started for the patient diagnosed with leukocytoclastic vasculitis. An increase was observed in the lesions of the patient during follow-up. It was learned that similar lesions were observed on the anterior parts of tibias previously for 2 times and she was diagnosed with vasculitis after biopsy performed. It was stated that the reason of vasculitis developing in the patient was determined to be brucellosis in both of attacks and cure was achieved after treatment for brucellosis. In her personal history, patient had complaints of muscle and joint paints, malaise, chill, shivering, and rash lasting about 2 months. When the risk factors for* Brucella* were examined, it was learned that the patient consumes fresh cheese.

In laboratory, the following were determined: white cell count: 8400/*μ*L, erythrocyte sedimentation rate: 50 mm/h (0–15), C Reactive Protein: 26,3 mg/L (<5), RF: 14 IU/mL (<14). The Rose Bengal test was positive. Standard tube agglutination (STA) test for* Brucella* was positive at titers of 1/320 and Coombs anti-*Brucella* test was positive at titers of 1/640. Also ELISA test for* Brucella* was positive for IgM and IgG antibodies. The patient was given a combination therapy of rifampicin (1 × 600 mg/day, po) and doxycycline (2 × 100 mg/day, po). It was determined that lesions were regressed at first week visit during follow-up. Rifampicin discontinued due to rifampicin-induced hepatotoxicity and ciprofloxacin therapy was added. A complete cure was achieved in the clinic of the patients at the end of 6-week treatment.

## 3. Discussion

Cutaneous manifestations observed in brucellosis were described in 1940 [4]. The cutaneous manifestations of brucellosis may develop due to direct inoculation, hypersensitivity phenomena, deposition of immune complexes, and direct invasion of the skin or via a hematogenous route of spread by the organism [5–7]. The cutaneous manifestations in brucellosis may be encountered as erythema, papules, petechiae, urticaria, impetigo, eczematous rash, erythema nodosum, subcutaneous abscess, and cutaneous vasculitis [1, 2]. Skin involvement was reported in 0,4 and 17% of the patients with brucellosis [3].

In the study performed by Ariza et al. and investigating 436 patients of brucellosis, the authors determined the most commonly observed cutaneous lesions to be disseminated violet erythematous, papulonodular eruption, and erythema nodosum-like lesions and they reported that these lesions were seen in about 6% of the patients [5]. In the study performed by Akcali et al. and investigating 140 patients of brucellosis, the authors determined skin involvements associated with brucellosis in a total of 8 patients (5,7%) as follows: 2 maculopapular eruptions, 2 erythema nodosum-like lesions, 1 psoriasiform lesion, 1 palmar erythema, 1 malar eruption, and 1 palmar eczema [8]. Artuz et al. determined the most commonly observed cutaneous lesions in the patients with brucellosis to be erythema nodosum and Metin et al. determined them to be urticaria-like papules [6, 9].

Vasculitic lesions associated with brucellosis were reported less frequently [4]. Yrivarren and Lopez described cryoglobulinemia and cutaneous vasculitis in three patients with brucellosis [10]. Immunological mechanisms play an important role in the pathogenesis of human brucellosis [6]. Hermida Lazcano et al. [11] reported a case of mixed cryoglobulinemia with renal failure, cutaneous vasculitis, and peritonitis due to* Brucella melitensis* and the authors concluded that all of the clinical picture (renal, hepatic, and cutaneous) was mediated by the cryoglobulins and/or caused by cryoglobulins. Dizbay et al. [12] determined hypocomplementemia, increased levels of polyclonal immunoglobulins (IgG, IgA, and IgE), positivity of rheumatoid factor and P-ANCA in the patients with renal failure, and leukocytoclastic vasculitis due to brucellosis and the authors stated that they thought that this picture was caused by mixed cryoglobulinemia. Similarly, also presence of hypocomplementemia, increased levels of polyclonal immunoglobulins, positivity of rheumatoid factor, and recurrent attacks of vasculitis in our case strongly suggest an underlying immunological abnormality.

Franco Vicario et al. [13] described a case of brucellosis presenting with granulomatous vasculitis in a 29-year-old female patient. Nagore et al. [14] and Karaali et al. [15] reported cases of brucellosis presenting with leukocytoclastic vasculitis and the lesions regressed in both of the cases within 48 hours after starting treatment for brucellosis. Similarly, also in our case, the disease exhibited itself as skin involvement and a rapid regression was determined in the present lesions with treatment started after diagnosis of brucellosis.

Vasculitic lesions are reported less frequently in skin involvement due to brucellosis. To the best of our knowledge, a case of brucellosis with recurrent attacks of vasculitis is described for the first time. Skin involvement and vasculitic lesions as a finding of skin involvement are nonspecific in brucellosis. Therefore, in the regions like Turkey where brucellosis is endemic, brucellosis should be kept in mind necessarily in the differential diagnosis of vasculitis.

## References

[1] Young E. J., Mandell G. L., Bennett J. E., Dolin R. (2005). *Brucella* species. *Principles and Practice of Infectious Disease*.

[2] Doganay M., Mese Alp E., Topcu A. W., Soyletir G., Doganay M. (2008). Bruselloz. *Infeksiyon Hastalıkları ve Mikrobiyolojisi*.

[3] Buzgan T., Karahocagil M. K., Irmak H. (2010). Clinical manifestations and complications in 1028 cases of brucellosis: a retrospective evaluation and review of the literature. *International Journal of Infectious Diseases*.

[4] Milionis H., Christou L., Elisaf M. (2000). Cutaneous manifestations in Brucellosis: case report and review of the literature. *Infection*.

[5] Ariza J., Servitje O., Pallarés R. (1989). Characteristic cutaneous lesions in patients with brucellosis. *Archives of Dermatology*.

[6] Metin A., Akdeniz H., Buzgan T., Delice I. (2001). Cutaneous findings encountered in brucellosis and review of the literature. *International Journal of Dermatology*.

[7] Berger T. G., Guill M. A., Goette D. K. (1981). Cutaneous lesions in brucellosis. *Archives of Dermatology*.

[8] Akcali C., Savas L., Baba M., Turunc T., Seckin D. (2007). Cutaneous manifestations in brucellosis: a prospective study. *Advances in Therapy*.

[9] Artuz F., Oram Y., Lenk N. (1994). Skin lesions of patients with brucellosis. *Turkish Journal of Dermatology*.

[10] Yrivarren J. L., Lopez L. R. (1987). Cryoglobulinemia and cutaneous vasculitis in human brucellosis. *Journal of Clinical Immunology*.

[11] Hermida Lazcano I., Méndez L. S., Santos J. S. (2005). Mixed cryoglobulinemia with renal failure, cutaneous vasculitis and peritonitis due to *Brucella melitensis*. *Journal of Infection*.

[12] Dizbay M., Hizel K., Kilic S., Mutluay R., Ozkan Y., Karakan T. (2007). Brucella peritonitis and leucocytoclastic vasculitis due to *Brucella melitensis*. *Brazilian Journal of Infectious Diseases*.

[13] Franco Vicario R., Balparda J., Santamaria J. M. (1985). Cutaneous vasculitis in a patient with acute brucellosis. *Dermatologica*.

[14] Nagore E., Sánchez-Motilla J. M., Navarro V., Febrer M. I., Aliaga A. (1999). Leukocytoclastic vasculitis as a cutaneous manifestation of systemic infection caused by *Brucella melitensis*. *Cutis*.

[15] Karaali Z., Baysal B., Poturoglu S., Kendir M. (2011). Cutaneous manifestations in Brucellosis. *Indian Journal of Dermatology*.

